# A tribute to William S. Sly (1932–2025)

**DOI:** 10.1172/JCI197698

**Published:** 2025-08-01

**Authors:** Ali Shilatifard, Stuart Kornfeld

**Affiliations:** 1 Northwestern University Feinberg School of Medicine, Chicago, Illinois, USA.; 2 Washington University School of Medicine in St. Louis, St. Louis, Missouri, USA.

Dr. William (Bill) S. Sly, an eminent physician-scientist known for his pioneering work in medical genetics and biochemistry, passed away on May 26, 2025. He dedicated his career to advancing the understanding and treatment of rare inherited metabolic disorders, particularly lysosomal storage disorders, and wrote the book (*The Metabolic and Molecular Bases of Inherited Disease*) on the subject (1).

Bill was born on October 19, 1932, in East St. Louis, Illinois. After graduating high school, Bill felt a divine calling, so he joined a Catholic seminary in St. Louis intending to become a priest. While he was there, in between prayers and pondering the mysteries of the universe, he enrolled in a few science courses. That’s where he discovered something truly *miraculous*: that elemental sodium reacts with water in a spectacular, explosive, science-fair-meets-action-movie kind of way. Now, most people would take that information, file it under “cool facts,” and move on. But not Bill. No, Bill was a man of action... So, one fine afternoon, inspired by a combination of curiosity, chemistry, and perhaps divine mischief, Bill marched over to the seminary’s peaceful fishpond — a tranquil oasis watched over by koi and maybe even a few confused ducks — and tossed in a chunk of sodium. What happened next was less “miracle of the loaves and fishes” and more “Michael Bay Presents: The Baptism of Sodium.” There was a loud boom, a splash, some steam, and possibly a fish that briefly achieved low Earth orbit. Bill, understandably proud of his experiment, didn’t anticipate how unamused the Rector would be. Summoned to the Rector’s office (a.k.a. “the confessional of consequences”), Bill was gently but firmly informed that perhaps his true calling wasn’t the priesthood after all. He was advised — politely, but urgently — to consider giving college a try instead.

Bill entered Saint Louis University, earning his undergraduate science degree in 1953 and his medical degree from the SLU School of Medicine in 1957, graduating at the top of his class. He completed his internal medicine training at Washington University in St. Louis and furthered his research experience at the National Institutes of Health, the French National Centre for Scientific Research in Paris, and at the University of Wisconsin–Madison. In 1964 he joined the faculty at Washington University in St. Louis, where he directed the Division of Medical Genetics for 20 years.

At Washington University, Bill and his team reported the first patient with a novel form of the lysosomal disorder mucopolysaccharidosis VII (MPS VII), which would become known as Sly syndrome. He established that the disease was due to a deficiency of β-glucuronidase activity (2). This was followed by a long effort to develop a treatment for this genetic disease, including studies that established that β-glucuronidase bound with high affinity to a cell-surface receptor on target cells, followed by internalization and delivery to lysosomes (3). He went on to show that the binding was mediated by mannose 6-phosphate residues on Asn-linked glycans on the β-glucuronidase (4). This was the first definitive evidence that glycans can serve as recognition molecules in biologic processes (5). Bill then worked with Ultragenyx Pharmaceutical to successfully develop β-glucuronidase for use in enzyme replacement therapy for Sly syndrome (6).

He also made significant contributions to the understanding of carbonic anhydrase deficiencies and hereditary hemochromatosis (7, 8). In recognition of his exceptional contributions to science, Bill was elected to the National Academy of Sciences and received numerous awards, including the Passano Foundation Award (shared with author Stuart Kornfeld), the Coriell Medal, the Marcel Simon Prize, the Peter Raven Lifetime Achievement Award, and many others.

In 1984, Bill took the helm as the third Chairman of the Edward A. Doisy Department of Biochemistry and Molecular Biology at the SLU School of Medicine. His interview for the position turned out to be more stressful than he had expected. The night before the interview, Bill realized that he had left his slide carousel on the plane coming back from a lecture at Harvard. With no slides to present at the interview, he gave a chalk talk about his work from memory. This apparently impressed everyone present, as he was offered the position that he held for the next 26 years. During this time, he hired numerous faculty members (including author Ali Shilatifard) and built a strong department. Many of his junior faculty have gone on to have very successful careers, with some serving as department chairs and others in executive positions at top academic institutions in the United States. The success of these hires directly correlates with Bill’s ability to identify scientific talents and mentor them to success.

Bill was a superb boss who embodied leadership well beyond administrative duties. He served as a steady, empathetic figure, a guiding force marked by unwavering support, compassion, and genuine care for his faculty’s success. We would say that he led with a fatherly presence — his actions consistently reflected a dedication to and a deep concern for the personal and professional growth of his department, offering mentorship and the resources needed to thrive. He was also gifted with the ability to skillfully impart personal advice, including some that he passed on from his own mentor. Ali clearly remembers when, as a new faculty member, he was at home one weekend putting up a fence; Bill told him something that went like this: “Ali, you can hire people to put up a fence, but you can’t hire people to use your talent to run your lab. You should be in the lab working on your science.”

In addition to being a prolific scientist and tremendous leader, Bill was also an amazing husband, father, and grandfather. He and his beloved wife of 63 years, Margaret Ann (Peggy), have 7 children, 26 grandchildren, and 7 great-grandchildren (Figures 1 and 2). Both Ali and Stuart had the honor of attending Bill’s memorial Mass at Our Lady of Lourdes parish in St. Louis and meeting his family, an exceptional group and a remarkable reflection of Bill himself.

Bill’s legacy will endure through his scientific contributions and in the many lives he touched throughout his distinguished career, within his community, and in the care of his beloved family. It is nearly impossible to summarize a rich life like that of Dr. Bill Sly, let alone give him the full recognition he deserves. He wouldn’t have wanted it any other way, as he truly embodied St. Louis University’s mottos, “Men and Women for Others” and AMDG — *Ad Majorem Dei Gloriam* (For the Greater Glory of God). Rest in peace, Bill. Your amazing legacy and contributions to science and humanity live on.

## Figures and Tables

**Figure 1:**
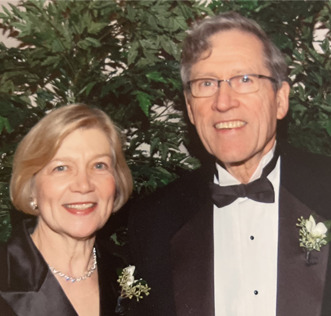
Bill and Peggy Sly.

**Figure 2:**
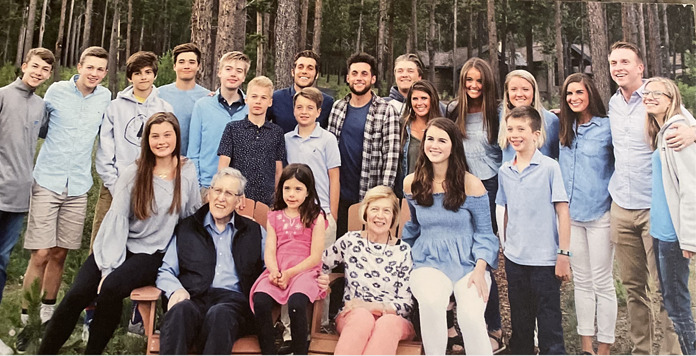
Bill and Peggy Sly with their grandchildren.

## Version 1 08/01/2025

Electronic publication
